# URAT1 inhibition in hyperuricemia and gout: from transporter biology to clinical precision therapy

**DOI:** 10.3389/fimmu.2026.1915148

**Published:** 2026-08-17

**Authors:** Guangtao Li, Yu Wang, Zhuoli Zhang

**Affiliations:** Department of Rheumatology and Clinical Immunology, Peking University First Hospital, Beijing, China

**Keywords:** benzbromarone, dotinurad, gout, hyperuricemia, lesinurad, URAT1 inhibitors, urate transporter 1

## Abstract

Impaired renal urate excretion is a major mechanism underlying hyperuricemia and gout, with urate transporter 1 (URAT1), encoded by SLC22A12, playing a central role in proximal tubular urate reabsorption. This review summarizes the biological relevance of URAT1, the pharmacological evolution of URAT1 inhibitors, and their clinical implications in urate-lowering therapy. Evidence from transporter biology, structural pharmacology, pharmacokinetic and pharmacodynamic studies, and clinical trials was narratively synthesized. URAT1 inhibitors lower serum urate by blocking renal tubular urate reabsorption and increasing urinary urate excretion, providing a mechanism complementary to xanthine oxidase inhibition. Early uricosuric agents established the clinical value of this approach but are limited by non-selective transporter inhibition, tolerability concerns, drug–drug interactions, and organ-specific safety issues. Newer selective URAT1 inhibitors have been developed to improve transporter selectivity, pharmacodynamic precision, and clinical usability. Current evidence supports selective URAT1 inhibition as an effective strategy for achieving serum urate targets, particularly in underexcretion-type hyperuricemia, while renal monitoring and prevention of uric acid stone formation remain important. Emerging agents may further expand treatment options, but long-term renal, hepatic, and cardiovascular safety require further validation. Overall, URAT1 inhibition represents a rational and increasingly precise therapeutic strategy for hyperuricemia and gout, with future research needed to define its long-term outcomes, comparative effectiveness, pharmacogenomic predictors, and broader cardio-renal-metabolic implications.

## 1 Introduction

The global prevalence of hyperuricemia and gout has increased markedly in recent decades, driven by dietary shifts, aging populations, and comorbidities such as chronic kidney disease and metabolic syndrome (1, 2). Hyperuricemia results from an imbalance between urate production and excretion, with impaired renal urate handling accounting for most cases (3, 4). Current urate-lowering therapies include xanthine oxidase inhibitors (XOIs), uricosuric agents, and recombinant uricase (5, 6), however, inadequate response, adverse effects, and contraindications limit their broad applicability (6, 7).

URAT1 (urate transporter 1), encoded by the SLC22A12 gene, located on the apical membrane of renal proximal tubular epithelial cells, mediates approximately 90% of renal urate reabsorption and plays a central role in urate homeostasis (8, 9). Beyond the kidney, URAT1 is expressed in the choroid plexus, vascular endothelium, placenta, hepatocytes, and adipocytes, suggesting broader roles in systemic urate distribution and signaling (10). Emerging evidence links URAT1 activity to blood pressure regulation and oxidative stress pathways, positioning it as a potential therapeutic target not only in gout but also in cardio-renal-metabolic disorders (10, 11). In gout specifically, serum urate lowering is mechanistically linked to the prevention of monosodium urate crystal formation and the reduction of crystal-driven innate immune activation, which strengthens the relevance of URAT1-directed therapy in an immunology-oriented context.

While XOIs reduce urate production, URAT1 inhibitors enhance renal excretion, offering a complementary mechanism particularly suitable for underexcretion-type hyperuricemia, which is highly prevalent in East Asian populations (11, 12). In this review, selective URAT1 inhibition refers to preferential blockade of URAT1 with limited inhibition of other clinically relevant urate or organic anion transporters, particularly OAT1, OAT3, and ABCG2. Consequently, URAT1 has become a key pharmacological target, with inhibitors evolving from early non-selective uricosurics to highly selective modern agents, alongside continued exploration of natural product–derived compounds. This review summarizes the pathophysiological relevance of URAT1 and the evolution of URAT1-directed therapy, with particular emphasis on dotinurad as the most clinically developed selective URAT1 inhibitor.

### 1.1 Epidemiology and rationale for URAT1 targeting

**Figure 1:**
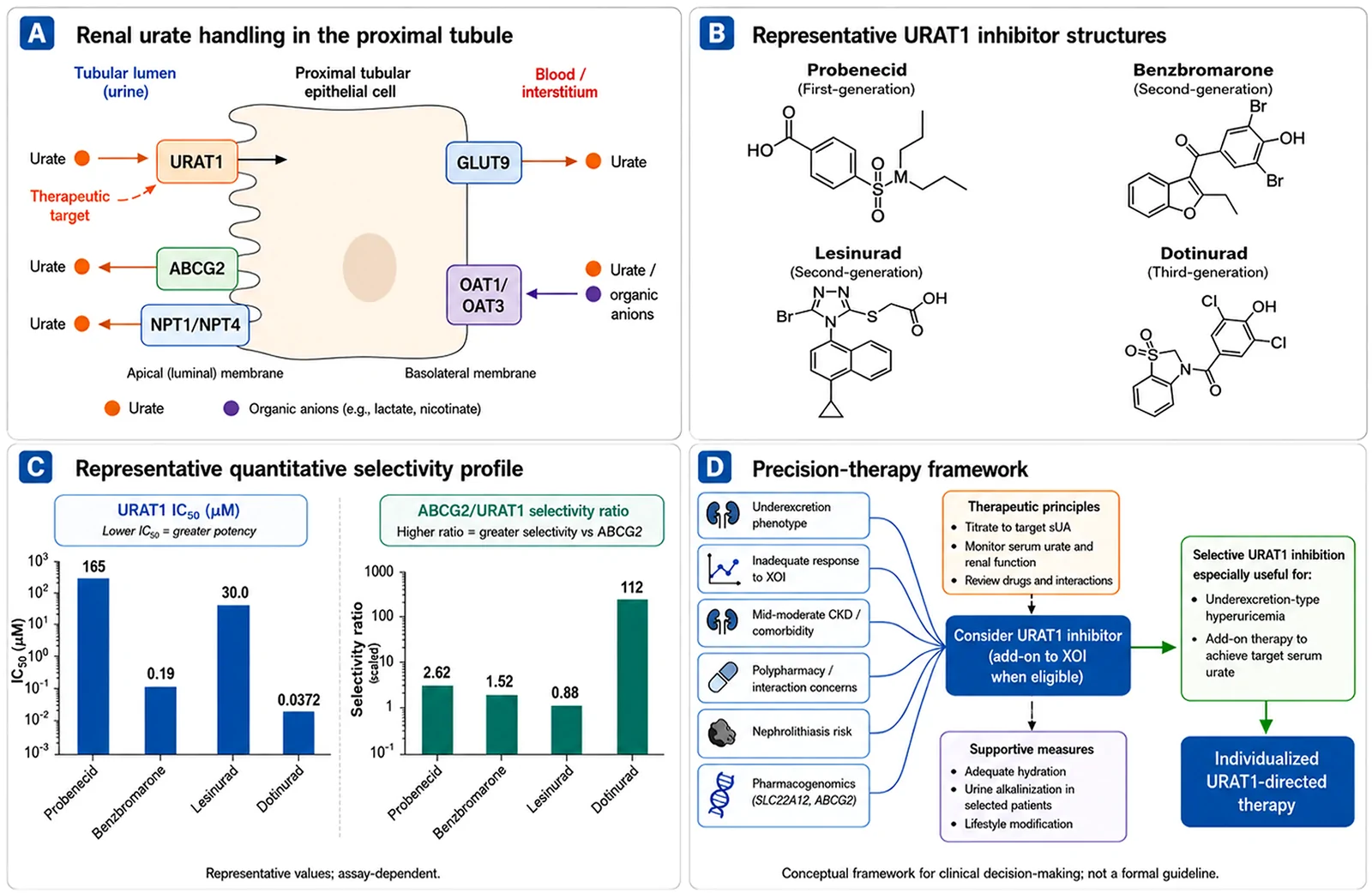
URAT1-directed therapy: renal urate handling, drug evolution, selectivity, and a precision-therapy framework. **(A)** Schematic overview of renal urate handling in the proximal tubule, highlighting URAT1 as a therapeutic target among major urate transporters. **(B)** Representative chemical structures of four URAT1 inhibitors across successive generations: probenecid (first-generation), benzbromarone and lesinurad (second-generation), and dotinurad (third-generation). **(C)** Representative quantitative comparison of URAT1 IC_50_ values and ABCG2/URAT1 selectivity ratios for selected agents. These values are assay-dependent and are shown for conceptual comparison. **(D)** A precision-therapy framework illustrating how clinical phenotype, comorbidity profile, and emerging pharmacogenomic considerations may inform URAT1-directed treatment selection.

Uric acid, the end product of purine metabolism, is primarily eliminated via the kidney (8, 9). Approximately two-thirds of urate excretion occurs renally, with the remainder through the intestine (10). Renal urate handling involves filtration, reabsorption, secretion, and post-secretory reabsorption mediated by transporters including URAT1, GLUT9, OAT1/3, and ABCG2 (11). Among these, URAT1 is the principal mediator of proximal tubular urate reabsorption (13) (**Figure 1**).

XOIs such as allopurinol and febuxostat reduce urate synthesis but frequently fail to achieve target serum urate levels in underexcretion-type patients (7), and may cause hypersensitivity or hepatic/renal toxicity (14–16). In contrast, uricosuric agents directly enhance renal urate excretion by inhibiting transporters such as URAT1 (17).

Beyond urate transport, URAT1 has been implicated in oxidative stress modulation and endothelial dysfunction, linking hyperuricemia to cardiovascular risk (18, 19). Therefore, targeting URAT1 may offer therapeutic benefits beyond urate lowering, supporting its continued prioritization in drug development pipelines.

### 1.2 Physiological and pathological role of URAT1

URAT1, encoded by SLC22A12 on chromosome 11q13, belongs to the organic anion transporter family and contains 12 transmembrane domains (20). It functions as an anion exchanger, mediating urate reabsorption in exchange for intracellular organic anions such as lactate and nicotinate.

Loss-of-function mutations in SLC22A12 cause familial renal hypouricemia, whereas gain-of-function variants or regulatory polymorphisms are associated with hyperuricemia (21). Genome-wide association studies confirm that SLC22A12 variants significantly influence serum urate levels and gout susceptibility across populations (22).

Excessive URAT1-mediated reabsorption contributes to hyperuricemia, monosodium urate crystal deposition, and gout flares, and may also promote nephropathy and cardiovascular complications (11).

Notably, ethnic differences in SLC22A12 polymorphisms have been documented (23), potentially affecting drug response and safety, underscoring the importance of pharmacogenomic considerations.

### 1.3 URAT1-directed urate lowering and the autoinflammatory biology of gout

Gout is a crystal-induced autoinflammatory disease in which persistent hyperuricemia promotes the formation and tissue deposition of monosodium urate (MSU) crystals. Once deposited, MSU crystals activate innate immune pathways, particularly in monocytes and macrophages, through NLRP3 inflammasome activation, caspase-1 activation, and the subsequent release of interleukin-1β. IL-1β signaling also promotes neutrophil recruitment and amplifies the acute inflammatory response characteristic of gout flares (24). Thus, the clinical relevance of urate-lowering therapy extends beyond biochemical correction of serum urate, because sustained urate lowering reduces the crystal burden that drives recurrent inflammation (24, 25).

The primary pharmacologic action of URAT1 inhibitors is inhibition of renal tubular urate reabsorption rather than direct suppression of inflammatory signaling (24, 26). By lowering serum urate and promoting dissolution of pre-existing deposits over time, effective urate-lowering therapy can reduce MSU crystal burden and is therefore expected to decrease recurrent flares and chronic crystal-associated inflammation (25). However, initiation or dose escalation of urate-lowering therapy may transiently increase the frequency of gout flares, which underscores the importance of gradual dose titration, appropriate flare prophylaxis, and patient counseling during treatment initiation (27–29). From this perspective, URAT1-directed therapy represents an upstream, disease-modifying strategy that complements, but does not replace, anti-inflammatory treatment within the broader immunopathology of gout (24–26).

### 1.4 Mechanisms of URAT1 inhibitors

URAT1 inhibitors reduce serum urate by blocking proximal tubular urate reabsorption, thereby increasing uricosuria. As an anion exchanger, URAT1 can be inhibited through substrate-site or allosteric binding, preventing urate translocation (30).

Earlier uricosuric agents such as probenecid and benzbromarone are not selective for URAT1. Probenecid inhibits URAT1 together with other renal organic anion transporters, whereas benzbromarone also interacts with multiple urate-handling transporters, which may influence both efficacy and safety (6). By contrast, newer agents such as dotinurad demonstrate substantially greater specificity for URAT1, thereby potentially reducing off-target effects and transporter-mediated drug interactions (31).

Combination therapy with URAT1 inhibitors and XOIs provides dual-pathway control of serum urate by simultaneously reducing production and enhancing elimination. This strategy offers additive or synergistic effects and is particularly valuable for patients inadequately controlled with monotherapy or those with persistent cardio-metabolic risk (6, 7, 32).

## 2 Evolution of URAT1 inhibitors and clinical research progress

The therapeutic development of URAT1 inhibitors has progressed substantially alongside advances in molecular pharmacology and renal urate transport physiology (33). In this review, the term “generation” is used as a pragmatic pharmacologic-historical framework rather than a strict regulatory label. First-generation agents refer to classical uricosurics developed before the molecular characterization of URAT1 and characterized by broad transporter inhibition. Second-generation agents include more potent or mechanistically refined uricosurics that still retain clinically relevant limitations related to off-target transport, safety, or restricted use. Third-generation agents refer to highly selective URAT1 inhibitors designed to improve transporter specificity, pharmacodynamic precision, and overall tolerability (7, 32).

First-generation agents, such as probenecid and sulfinpyrazone, act non-selectively on multiple renal transporters and are limited by tolerability concerns and reduced efficacy in renal impairment (6). Second-generation agents, including benzbromarone and lesinurad, improved potency or mechanistic refinement, but important safety and regulatory limitations remained (34–36). Third-generation agents are typified by highly selective URAT1 inhibitors such as dotinurad, which were designed to improve transporter specificity and clinical usability (26, 37, 38).

Overall, the evolution of URAT1 inhibitors illustrates a shift from broad-spectrum uricosuric therapy toward more transporter-selective modulation with a potentially improved therapeutic index (**Table 1**). This trend may broaden the applicability of uricosuric therapy in selected patients, but its clinical implications still depend on the strength and generalizability of the available evidence.

**Table 1:** Representative first- to third-generation URAT1 inhibitors: pharmacologic and clinical features.

Generation	Agent	Main transporter profile / mechanism	Representative URAT1 IC50 (μM)	Representative selectivity ratio vs URAT1 (ABCG2 / OAT1 / OAT3)	Representative clinical evidence	Typical use / cautions
First generation	Probenecid (6, 8, 26)	Broad uricosuric; inhibits URAT1, OAT1, OAT3, and GLUT9	165	2.62 / 0.0661 / 0.0144	Early monotherapy studies; mean treatment duration approximately 12 weeks; target sUA ≤6 mg/dL achieved in approximately 33%.	Best in patients with preserved renal function; interaction potential.
Second generation	Benzbromarone (26, 34, 36)	Potent URAT1 inhibition; secondary GLUT9 inhibition; may reduce urate-induced oxidative stress.	0.19	1.52 / 16.5 / 5.09	14-week double-blind studies; target sUA achievement up to 83.6%; benefit also reported in underexcretion-type hyperuricemia.	Useful in mild–moderate CKD; monitor liver function because of hepatotoxicity concern.
Second generation	Lesinurad (8, 26, 36–38)	Inhibits URAT1; also interacts with OAT4 and OAT10; approved as add-on therapy to XOI.	30.0	0.880 / 0.233 / 0.0357	CLEAR studies; with allopurinol, target sUA ≤6 mg/dL in approximately 55.4–66.5% versus approximately 23.3% with control; no clear flare benefit.	Add-on therapy in inadequate XOI responders; renal adverse events limited broader use.
Third generation	Dotinurad (8, 26, 31, 39, 40)	Highly selective URAT1 inhibitor with minimal inhibition of ABCG2 and OAT1/3.	0.0372	112 / 110 / 35.5	14-week Japanese phase 3 studies: target sUA up to 86.2%; Chinese phase 3 study: week 24 target sUA 73.6% versus 38.1% with febuxostat 40 mg/day.	Broad potential use, including mild–moderate CKD; continue renal monitoring and stone-prevention measures.

URAT1, urate transporter 1; sUA, serum urate; XOI, xanthine oxidase inhibitor; CKD, chronic kidney disease.

Representative IC50 and selectivity ratios are compiled from original pharmacology studies and review sources; exact values may vary by assay conditions.

Representative clinical evidence summarizes selected studies and should not be interpreted as direct head-to-head comparison across agents.

### 2.1 First-generation URAT1 inhibitors: early uricosuric agents

The first-generation uricosuric agents, including probenecid and sulfinpyrazone, were historically developed before the molecular characterization of URAT1 (39). Their primary mechanism involves the inhibition of urate reabsorption in the renal proximal tubule, leading to increased urinary uric acid excretion. Probenecid remains one of the earliest approved agents and demonstrated a significant serum urate (sUA) reduction from 6.1 mg/dL to 3.4 mg/dL over a 4-week treatment period in clinical trials (6). Long-term use was associated with a notable decrease in gout flare frequency from 3.6 episodes per year to less than one. However, probenecid’s effectiveness is highly dependent on preserved renal function and is not recommended in patients with moderate-to-severe chronic kidney disease (CKD). Adverse effects such as rash and gastrointestinal discomfort are relatively common, although severe hepatotoxicity is rare (6). Sulfinpyrazone, another early uricosuric agent, lowers serum urate primarily by inhibiting renal urate reabsorption. Its contemporary use is limited by restricted availability, limited modern efficacy and safety data, renal safety concerns, and the potential for clinically relevant drug–drug interactions (6, 40).

### 2.2 Second-generation URAT1 inhibitors: improved potency and clinical challenges

In response to the limitations of earlier agents, second-generation URAT1 inhibitors were developed with enhanced potency and more acceptable safety profiles. Benzbromarone, a coumarin derivative, exhibits potent URAT1 inhibition and remains widely used in parts of Asia and South America (41). It demonstrated superior efficacy over allopurinol in achieving sUA targets, even in patients with moderate renal impairment (35). Nevertheless, benzbromarone’s global adoption has been limited by historical concerns over rare but potentially fatal hepatotoxicity. Despite this, no clear evidence of a dose-dependent or predictable hepatotoxic mechanism has been established, and post-marketing surveillance suggests an acceptable risk-benefit ratio when liver function is appropriately monitored (36).

Lesinurad represents a milestone as the first approved selective URAT1 inhibitor (37). Approved in 2015 for use in combination with XOIs, lesinurad significantly improved target sUA achievement compared to allopurinol monotherapy in the CLEAR 1 and CLEAR 2 trials (36, 38). At 200 mg daily, lesinurad achieved sUA <6 mg/dL in 55.4% to 66.5% of patients versus 23.3% in the control group (P < 0.0001) (36). However, no significant benefit was observed in secondary outcomes such as flare reduction or tophus resolution. Importantly, higher doses (400 mg daily) were associated with increased renal adverse events, including serum creatinine elevation and acute kidney injury. These concerns led to a recommendation of 200 mg for combination use and ultimately to market withdrawal in 2019 due to commercial limitations and regulatory complexities (36).

### 2.3 Third-generation URAT1 inhibitors: advanced selectivity and clinical efficacy

The third generation of URAT1 inhibitors represents a significant pharmacological advancement, characterized by superior transporter selectivity and a refined safety profile. Dotinurad, the most extensively validated compound in this class, exemplifies these improvements through its highly specific inhibition of URAT1 and minimal interference with off-target transporters such as ABCG2 and OAT1/3 (26).

Structurally, dotinurad was developed as a highly selective URAT1 inhibitor with a scaffold distinct from earlier uricosurics. Available clinical studies have not identified a clear hepatotoxicity signal, although continued long-term observation remains important (41–43). In addition, structural and mutagenesis studies indicate that dotinurad binds within the URAT1 transport pocket and stabilizes a non-conductive conformation through interactions involving K393, R477, and surrounding hydrophobic residues (31, 34).

In comparative transporter assays, dotinurad inhibited URAT1 with an IC_50_ of 0.0372 μM, compared with 0.190 μM for benzbromarone, 30.0 μM for lesinurad, and 165 μM for probenecid. The corresponding ABCG2-to-URAT1 IC_50_ ratios were 112, 1.52, 0.880, and 2.62, respectively, indicating substantially greater selectivity of dotinurad for URAT1 under the same assay conditions (26). These features translate into fewer off-target effects and reduced risk of renal or hepatic toxicity, particularly via inhibition of uremic toxin transporters such as ABCG2 and OAT1/3 (37). Notably, febuxostat has also been reported to inhibit ABCG2, a key efflux transporter for urate and uremic toxins. Inhibition of ABCG2 may aggravate renal and cardiovascular outcomes, particularly in patients with CKD or existing cardiovascular disease (44). The high selectivity of dotinurad for URAT1 over ABCG2 may thus offer additional safety advantages in this population. Dotinurad showed weak *in vitro* inhibition of major drug-metabolizing enzymes, suggesting a relatively low potential for clinically meaningful CYP-mediated drug–drug interactions; however, this conclusion is primarily based on nonclinical pharmacokinetic assessments (45). In an open-label, two-period study in healthy adult males, co-administration of oxaprozin with a single 4 mg dose of dotinurad increased dotinurad AUC_0_–inf by 16.5% and reduced the urinary excretion of its glucuronide conjugates by 34.3%, while Cmax was essentially unchanged. The 90% confidence intervals for the geometric mean ratios of the plasma pharmacokinetic parameters remained within the prespecified 0.80–1.25 range, and no clinically meaningful drug–drug interaction or new safety concern was identified (46). Available dose-ranging and phase 2/3 clinical studies indicate that dotinurad produces consistent serum urate lowering across the therapeutic dose range and is generally well tolerated in the studied populations (41–43). This saturation profile may help avoid excessive uricosuria and abrupt changes in urate handling. However, any putative link between intensive urate lowering and neurodegenerative disease remains unproven and should not be overstated in the present review. Moreover, dotinurad-induced urate excretion returns to baseline within 24–48 hours after administration, indicating a transient and physiologically manageable impact on renal urate handling. The renal clearance of urate achieved with dotinurad remains within the normal physiological range and is notably lower than that observed in patients with URAT1 loss-of-function mutations, supporting its renal safety. Collectively, these characteristics suggest that dotinurad provides effective urate-lowering therapy with a controlled rate of action. Nevertheless, nephrolithiasis and renal adverse events remain clinically relevant considerations during uricosuric therapy, and standard preventive measures such as adequate hydration, urine alkalinization in selected patients, and clinical monitoring remain important.

A pivotal phase III randomized, multicenter, double-blind trial conducted across 30 sites in China (NCT05007392) provided clinically meaningful evidence supporting the superior efficacy of dotinurad over febuxostat in the management of gout (47). Among 451 randomized patients, 441 were included in the full analysis set. At week 24, a significantly higher proportion of patients achieved the target sUA level of ≤6.0 mg/dL with dotinurad 4 mg/day than with febuxostat 40 mg/day (73.6% vs. 38.1%; adjusted difference 35.9%, 95% CI 27.4–44.4; p<0.0001). This superiority was confirmed by sensitivity analyses. Dotinurad 2 mg/day was also non-inferior to febuxostat 40 mg/day at week 12, with responder rates of 55.5% and 50.5%, respectively. The mean reduction in sUA at week 24 was greater with dotinurad than with febuxostat (45.9% vs. 30.6%), and the benefit was consistent across predefined subgroups. The safety profile of dotinurad was comparable to that of febuxostat. The incidence of treatment-emergent adverse events was similar between groups (90.6% vs. 90.2%), and severe or serious adverse events were infrequent, with no new safety signals identified. Events of special interest, including urinary calculi, renal-related adverse events, and liver-related adverse events, occurred at comparable rates between dotinurad and febuxostat. Overall, these findings support dotinurad as an effective and generally well-tolerated selective urate-lowering therapy in Chinese patients with gout. However, the comparative benefit should be interpreted in the context of regional treatment practice and the use of febuxostat 40 mg/day as the comparator dose. Because uricosuric therapy may increase the risk of uric acid stone formation, adequate hydration, urinary alkalization, and renal monitoring may be considered in high-risk patients.

Long-term efficacy and safety were evaluated in a Japanese multicenter, open-label, dose-escalation study lasting 34 or 58 weeks. At week 58, serum urate levels were reduced from baseline by 47.17% with dotinurad 2 mg and by 57.35% with dotinurad 4 mg. The proportions of patients achieving serum urate ≤6.0 mg/dL were 91.30% and 100.00%, respectively. The overall incidences of adverse events and adverse drug reactions were 65.2% and 21.8%, respectively. Serious adverse events occurred in 2.7% of patients, and a causal relationship with dotinurad could not be excluded for one case of stage I gastric cancer (48). After 58 weeks of treatment, the mean change in eGFR from baseline was minimal and not statistically significant (-1.1 ± 7.8 mL/min/1.73 m², p = 0.158) (48). A subsequent pooled analysis stratified patients by baseline renal function from stage G1 to G3b and found that eGFR remained generally stable during long-term dotinurad treatment. Although a statistically significant change was observed in the G2 subgroup at week 34, this difference was no longer significant at week 58, and no clinically relevant deterioration was observed in the G3a or G3b subgroups. These findings support the observed renal tolerability of dotinurad in patients with normal renal function and mild-to-moderate renal impairment, although the small number of patients with G3b disease warrants cautious interpretation (49). Hepatic parameters were also monitored during long-term treatment. Although statistically significant changes in AST or γ-GTP were observed at certain time points, the absolute changes were small and were considered to be within the range of normal physiological fluctuation. No clinically relevant persistent abnormality in AST, ALT, or γ-GTP was identified over the observed treatment period (48).

In a 2023 network meta-analysis including 12 randomized controlled trials (n = 1851), dotinurad 4 mg once daily ranked highly for achieving target serum urate levels among the evaluated uricosuric regimens; however, this finding was based on indirect comparisons across heterogeneous trials and should therefore be interpreted cautiously (50).

Emerging evidence has raised interest in possible metabolic and vascular effects of dotinurad beyond serum urate lowering. In preclinical models, dotinurad ameliorated insulin resistance, hepatic steatosis, and systemic inflammation in high-fat diet-fed mice (42). In a small exploratory clinical study, 24-week treatment was associated with improvements in arterial stiffness and oxidative stress markers. However, these findings remain preliminary and hypothesis-generating, and they should not be interpreted as established cardiovascular or metabolic outcome benefits (51). Despite its pharmacological advantages, the generalizability of dotinurad’s clinical evidence remains limited, as most available data are derived from Asian populations. Further studies in ethnically and geographically diverse cohorts are needed to clarify its efficacy, safety, and optimal clinical positioning across broader hyperuricemic and gout populations.

### 2.4 Investigational selective URAT1 inhibitors and other pipeline agents

Beyond clinically established agents such as dotinurad in Asia, several investigational selective URAT1 inhibitors remain in the development pipeline and aim to further optimize urate-lowering potency, combination utility, and dosing convenience.

#### 2.4.1 Verinurad (RDEA3170)

Verinurad (RDEA3170), a structural analog of lesinurad, is a highly potent URAT1 inhibitor. Phase II trials in the United States and Japan showed that verinurad monotherapy reduced serum urate by up to 55.8% at 12.5 mg. However, dose-dependent renal adverse events, including transient and reversible serum creatinine elevations, were observed. Although no irreversible renal injury has been reported, follow-up durations remain limited. These safety concerns and the absence of long-term renal outcome data have slowed clinical development. Combination therapy with febuxostat produced additive urate-lowering effects without increasing uricosuria, but long-term safety remains unclear. Development of verinurad is currently on hold pending resolution of these safety issues (52, 53).

#### 2.4.2 AR882 (pozdeutinurad)

Pozdeutinurad (AR882) is a potent, selective, once-daily oral URAT1 inhibitor. In a phase IIa study, AR882 monotherapy (50 mg daily) reduced serum urate by approximately 53%, outperforming allopurinol and febuxostat (54). In a randomized phase IIb trial involving 140 patients with gout, 12-week treatment with 50 or 75 mg reduced median serum urate from 8.6 mg/dL to 5.0 and 3.5 mg/dL, respectively; 78% and 89% of patients achieved serum urate <6 mg/dL (55). Pozdeutinurad is also being evaluated in two 12-month global phase III trials, REDUCE 1 and REDUCE 2 (NCT06846515, NCT06439602). Positive topline results have been reported from REDUCE 2, although detailed peer-reviewed data are not yet available. REDUCE 1 and the Chinese phase II/III febuxostat-controlled study have not yet reported results (NCT06846515, NCT06603142).

#### 2.4.3 Other novel URAT1 inhibitors and natural products

Ruzinurad sodium (SHR4640) was approved by the Chinese National Medical Products Administration in May 2026 for the treatment of gout accompanied by hyperuricemia. In its phase III trial involving 779 patients, sustained achievement of serum urate ≤360 μmol/L at week 16 was significantly higher with ruzinurad than with allopurinol (39.7% vs 26.5%; *P* < 0.0001), with a generally manageable safety profile (56). Parallel research on natural products has identified multiple flavonoids, phenolic acids, and alkaloids from medicinal plants with *in vitro* URAT1 inhibitory activity, providing structural templates for semi-synthetic drug development (57). Chen et al. comprehensively reviewed these natural compounds and proposed their potential as prototypes for future drug design (30). Although most remain preclinical, they offer molecular diversity and potentially favorable safety profiles.

In addition, evidence from regional studies should be interpreted with attention to differences in comparator regimens, approved dosing, and whether enrolled populations had gout or asymptomatic hyperuricemia.

## 3 Conclusion and future perspectives

The therapeutic landscape of URAT1 inhibition has evolved considerably over the past decades, driven by improved understanding of urate transporter biology and the unmet clinical needs in managing hyperuricemia and gout (**Table 1**). First- and second-generation uricosuric agents, although historically important, are limited by safety concerns such as hepatotoxicity and gastrointestinal intolerance, as well as by reduced efficacy in patients with renal impairment due to their non-selective inhibition of multiple renal transporters.

The introduction of dotinurad, a highly selective URAT1 inhibitor, represents an important step in the evolution of uricosuric therapy (50). Available clinical studies from Japanese and Chinese populations indicate that dotinurad provides effective serum urate lowering with a generally favorable safety profile (37, 38, 50, 52–54, 58), However, its place in global treatment algorithms still depends on broader validation across diverse populations and practice settings.

Despite this progress, several challenges and opportunities remain for further advancement. Future URAT1 inhibitors should strive to enhance selectivity and reduce off-target effects, particularly in polypharmacy settings common among patients with CKD, diabetes, and cardiovascular disease (42). Advances in structural biology, such as high-resolution cryo-EM imaging of URAT1, are likely to inform rational design of next-generation molecules with superior pharmacologic precision (59).

Beyond urate lowering, systemic effects of URAT1 inhibition are gaining attention. Evidence suggests potential metabolic and vascular benefits, including improvements in insulin sensitivity, lipid metabolism, and oxidative stress (51). Dotinurad has been associated with reduced arterial stiffness and inflammatory markers in hyperuricemic patients (51). However, large outcome trials are required to confirm effects on cardiovascular and renal endpoints.

Pharmacogenomic insights may also contribute to individualized therapy. Variants in urate-transporter genes, particularly SLC22A12 and ABCG2, are important determinants of urate handling and may contribute to interindividual variability relevant to treatment selection and response (22, 58). Ethnic variability in urate handling may partly explain differences in efficacy between Asian and Western cohorts (53), and future integration of genetic information into clinical algorithms may help optimize treatment selection and dosing strategies.

Additionally, oral fixed-dose combinations that pair URAT1 inhibitors with xanthine oxidase inhibitors (XOIs) or sodium-glucose cotransporter 2 (SGLT2) inhibitors are emerging as attractive therapeutic strategies. These combinations may offer synergistic urate-lowering effects while minimizing individual drug exposure, thus reducing toxicity and enhancing adherence (50).

Lastly, the collection of long-term real-world evidence is essential to validate the durability, safety, and cost-effectiveness of new URAT1-targeted therapies in routine practice. As promising investigational agents such as AR882 and verinurad advance, further clinical studies will be critical to defining their future role in treatment algorithms and guidelines (27, 53, 60).

Importantly, the current evidence base should be interpreted alongside prevailing international treatment recommendations (27, 60). Despite strong efficacy and safety data in Asian populations, dotinurad has not yet been approved in the United States or European Union, possibly due to limited phase III data in Western cohorts, genetic variability, and regulatory emphasis on long-term cardiovascular outcomes. Global multicenter trials may facilitate broader adoption.

This review has several limitations. First, it is a narrative rather than a systematic review. Although structured database searches were conducted, the potential for selection bias cannot be fully excluded. Second, several important clinical questions remain incompletely answered. These include the long-term real-world adherence and durability of URAT1 inhibitors beyond 58 weeks, particularly in patients with comorbidities. Finally, we acknowledge the importance of visual data representation; in response, we have incorporated additional figures and tables to summarize URAT1 inhibitor structures, clinical efficacy, and safety profiles.

In summary, the evolution of URAT1 inhibitors reflects a broader trend toward precision pharmacology in gout and hyperuricemia. Continued innovation, supported by translational research and personalized medicine approaches, holds the promise of safer, more effective, and metabolically beneficial urate-lowering strategies for a wide spectrum of patients.
